# Comparison of the value of ultrasound-guided fine needle aspiration biopsy and contrast-enhanced ultrasound in different sizes of thyroid nodules

**DOI:** 10.1097/MD.0000000000039843

**Published:** 2024-09-27

**Authors:** Qi Liu, Liquan Ouyang, Shengchu Zhang, Yuxia Yang

**Affiliations:** a Department of Thyroid and Breast Surgery, Geriatric Hospital Affiliated to Wuhan University of Science and Technology, Wuhan, China; b Department of Thyroid and Breast Surgery, Yichang Central People’s Hospital, Yichang, Hubei, China; c Department of Thyroid and Breast Surgery, The Second Affiliated Hospital of Zhejiang Chinese Medical University, Zhejiang, Hangzhou, China; d Department of Pathology, Yichang Central People’s Hospital, Yichang, Hubei, China.

**Keywords:** contrast-enhanced ultrasound, nodule size, thyroid cancer, thyroid microcarcinoma, thyroid nodules, thyroid tumors, ultrasound-guided fine needle aspiration biopsy

## Abstract

The purpose of this study was to evaluate the diagnostic value of ultrasound-guided fine needle aspiration biopsy (US-FNAB) and contrast-enhanced ultrasound (CEUS) in the presence of thyroid nodules of different sizes. We retrospectively analyzed 110 patients with surgically operated unimodular thyroid nodules in Yichang City Central Hospital from July 1, 2021, to April 1, 2023, all of whom underwent conventional thyroid ultrasound, CEUS, and US-FNAB, and all of whom were classified according to the size of nodules into <0.5, 0.5 to 1, and ≥1 cm groups. The diagnostic accuracy, sensitivity, and specificity of the 2 methods for benign and malignant nodules were calculated. Among 110 thyroid nodules, 102 were malignant nodules and 8 were benign nodules. In patients with nodule diameters <1 cm the sensitivity of US-FNAB and CEUS was 87.02% and 93.89%, respectively, and the specificity was 100.00% and 66.67%, with an accuracy of 87.31% and 93.28%, respectively. In patients with nodule diameter <0.5 cm, the sensitivities of US-FNAB and CEUS were 74.29% and 100.00%, respectively, and the differences were statistically significant (*P* < 0.05); in patients with nodule diameter 0.5 to 1 cm, the sensitivities of US-FNAB and CEUS were 79.59% and 95.92%, respectively, and the differences were statistically significant (*P* < 0.05); among patients with nodule diameters ≥1 cm, the sensitivities of US-FNAB and CEUS were 88.89% and 77.78%, respectively, with no statistically significant difference (*P* > 0.05). For thyroid nodules ≤1 cm in diameter, the sensitivity of CEUS examination was higher than that of US-FNAB; and CEUS still has good diagnostic accuracy in the diagnosis of benign and malignant thyroid nodules <0.5 cm, CEUS is recommended for thyroid nodules diagnosed negatively by US-FNAB with a diameter of <1 cm; and CEUS should be preferred for thyroid nodules with a diameter of <0.5 cm. CEUS should be preferred for thyroid nodules <0.5 cm in diameter.

## 1. Introduction

Studies have shown that thyroid nodules can be detected by plain US in 65% of the general population, 90% of which are benign and generally require no specific treatment; only about 10% of patients with thyroid nodules are at risk for malignancy.^[1]^ However, the incidence and mortality of thyroid cancer are still increasing worldwide.^[2]^ Papillary thyroid carcinoma (PTC) is the most common thyroid malignancy, accounting for about 85% of cases, with a good prognosis and a 20-year survival rate of about 90%.^[3]^ Papillary thyroid microcarcinoma (PTMC), defined as papillary thyroid carcinoma of 1 cm or less, has had the highest growth rate worldwide in recent years.^[2,4]^ Malignant nodules often have an early tendency to metastasize to the cervical lymph nodes, which affects the surgical prognosis and survival rate,^[5]^ so early diagnosis and differentiation of benign and malignant thyroid nodules are very important.

Fine-needle aspiration biopsy (FNAB) is now recognized as an accurate and cost-effective tool for identifying benign and malignant thyroid glands, which can clarify the pathological type of the tumor and provide a good match with postoperative pathology.^[6,7]^ US is the most important imaging tool for the evaluation of thyroid nodules; it can determine the number, size, morphology, and location of the nodules,^[8,9]^ and it is also excellent in detecting small nodules (<1 cm in diameter).^[10,11]^ The main advantage of CEUS is its ability to assess the sequence and intensity of vascular perfusion and hemodynamics within thyroid nodules,^[7]^ which can significantly improve the sensitivity and specific diagnostic compliance of thyroid nodules based on conventional US.^[12]^ Some studies have demonstrated that CEUS can greatly improve the diagnostic accuracy of PTMC due to its property of detecting perfusion within the nodule.^[13]^

The differential diagnostic value of US-FNAB and CEUS for thyroid nodules of different sizes has not yet been uniformly concluded, especially since the diagnostic value of CEUS in thyroid micronodules (<0.5 cm in diameter) has rarely been reported. In this article, we retrospectively analyzed the ultrasonographic features of 110 cases of pathologically confirmed thyroid nodules, to explore the diagnostic value of US-FNAB and CEUS in different sizes of thyroid nodules, and provide a reference for the early diagnosis of micronodules.

## 2. Materials and methods

### 2.1. Patient selection

110 cases of single thyroid nodules that underwent US-FNAB and CEUS and had post-surgical pathology results from July 1, 2021, to April 1, 2023, in our hospital were selected. Inclusion criteria were: patients who underwent routine US, CEUS, and US-FNAB and eventually underwent thyroid surgery, complete imaging and pathology data, no previous thyroid cancer, thyroid or parathyroid surgery, and no history of other malignant tumors in patients. Exclusion criteria: those who were allergic to contrast media and those with severe cardiopulmonary dysfunction. It included 102 patients with malignant nodules (85 females and 17 males; age range: 20–61 years old, mean 42.31 years old) and 8 patients with benign nodules (6 females and 2 males; age range: 33–63 years old, mean 49.57 years old), with a mean size of nodules of 0.73 centimeters (range 0.1–2.8 cm). Postoperative pathologic findings were used as the final diagnosis.

The study was approved by the Ethics Committee of Yichang Central Hospital (Approval No: 2022-104-01), and all patients had signed the relevant informed consent forms. Patient data is anonymised and all patient data is protected by encryption and secure storage.

### 2.2. CEUS technique

All US examinations were performed using a commercially available scanner (Resona R9s; Myriad, Shenzhen, China) with an ultrasound probe model L15-3WU, which can perform both routine US and CEUS examinations. The patient was placed in the supine position, and the neck was fully exposed after elevating the shoulders; the conventional US was performed first to observe the target nodules, and the bilateral lobes and isthmuses of the thyroid gland were examined, and a 2-dimensional high-frequency probe was used to carefully measure the patient’s thyroid nodules in terms of their size, location, number, Doppler flow signals, and other general characteristic information. The largest cross-section of the lesion was selected as the cross-section for US imaging, and the entire image of the thyroid nodule and the surrounding area were observed as much as possible. Then the appropriate image was selected to switch to CEUS mode. CEUS was performed at low acoustic intensity (low mechanical index 0.15). Sulfur hexafluoride microbubble contrast agent SonoVue (2.4 mL, BR1; Bracco SpA, Milan, Italy) was injected into the elbow vein followed by saline (5 mL). After injection, each image acquisition lasted 3 minutes, and images were automatically saved. During imaging, the probe was immobilized and the patient was asked to avoid swallowing. The entire dynamic imaging procedure was stored on the machine’s hard disk and the US workstation for subsequent memory processing and analysis. During imaging, the focal point of the US was placed at the same level, and various imaging conditions, including mechanical index (MI), depth, etc., were kept constant during the imaging process. Qualitative and quantitative data were recorded while observing the real-time US imaging process. The entire examination is fairly brief and can be completed in about 5 to 10 minutes.

Routine ultrasound diagnostic work is required to be performed by physicians with at least 3 years of experience in ultrasound diagnosis of thyroid diseases. For diagnostic imaging, 2 associate physicians were blinded to each other and gave independent diagnostic opinions. In case of disagreement, the chief physician is consulted for joint discussion and finalization of the diagnosis.

**Figure 1.**
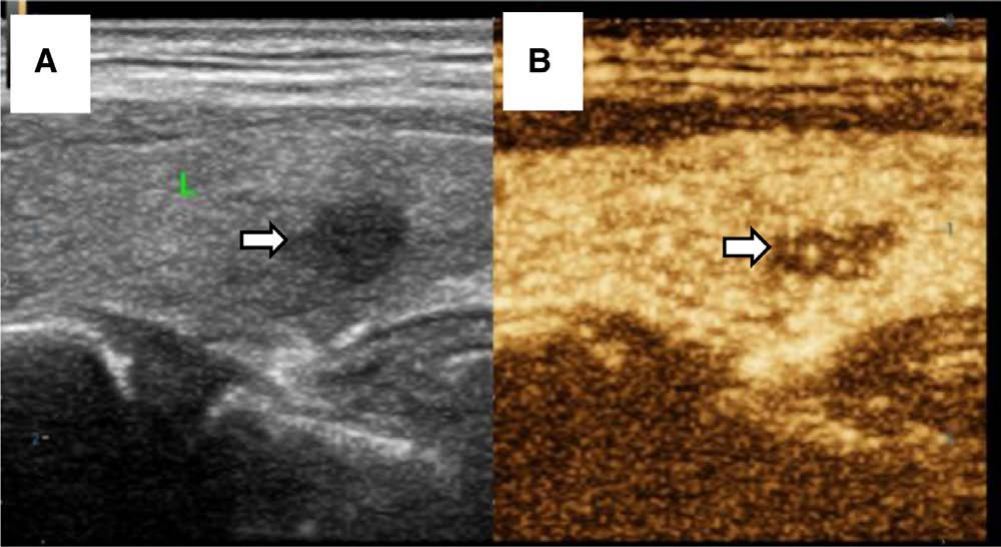
Male, 49 years old, dorsal hypoechoic nodule (0.7 × 0.4 cm) in the lower middle segment of the left lobe of the thyroid gland, with postoperative pathology suggestive of PTMC: (A) Gray-scale ultrasound showed a hypoechoic nodule with unclear borders; (B) During CEUS scanning, an inhomogeneous hypo-enhanced nodule appeared 9 seconds after injection of contrast medium. CEUS = contrast-enhanced ultrasound, PTMC = papillary thyroid microcarcinomar.

CEUS diagnosed malignant nodules with hypo-enhancement, incomplete or inhomogeneous enhancement, blurred margins, or irregular patterns (positive results), while the remaining features, such as homogeneous, iso-enhancement, or hyper-enhancement, were diagnosed as benign nodules (negative results; Fig. 1).

### 2.3. FNAB technique

**Figure 2.**
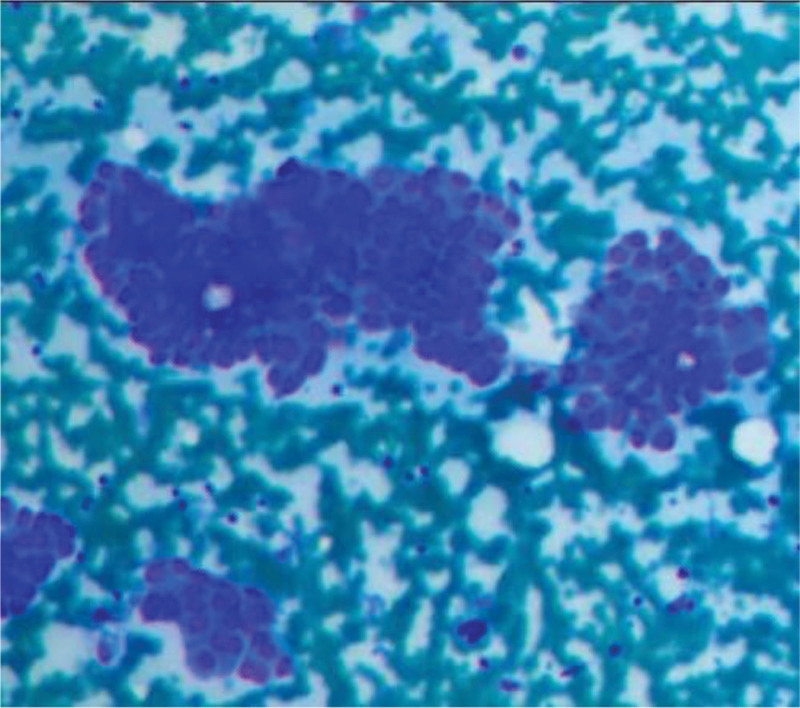
The cytological pathology was Bethesda III category, with postoperative pathology suggestive of PTC. Microscopically, adenoepithelioid cells were seen, with crowded and overlapping nuclei, enlarged ovoid nuclei, visible nucleoli, nuclear grooves, intranuclear pseudo-inclusions, and partially formed papillary structures (Diff-Quik stain, original magnification, ×100). PTC = papillary thyroid carcinoma.

Patients take a sitting position or supine position, expose the thyroid gland, ask the patient to swallow action, record the size, texture, and other relevant information of the mass after careful examination of the mass, local skin disinfection routinely, using disposable 5 mL sterile syringe with a 7-gauge needle, according to the finger pressure to determine the size, shape, and depth of the tumor, if it can’t be palpated, then it is necessary to examine the guidance of the US. Fix the mass, puncture it quickly, keep 0.5 mL of low negative pressure, rapidly reciprocate the needle suction 15 to 20 times, and then smear the needle suction material quickly. The action should be quick and gentle when smearing to reduce the blood content in the needle suction material and avoid the accumulation of cells due to the coagulation of blood and the difficulty of smearing, which would affect the observation of the smear and the correct diagnosis. Diff-Quick staining was used for light microscopic observation and diagnosis. The diagnosis was made by a senior cytopathologist, and the fine needle aspiration biopsy (FNA) puncture specimens were classified as negative for inconclusive, benign, or atypical lesions, and positive for follicular tumor or malignant tumor cells (Fig. 2).

### 2.4. Postoperative pathology

**Figure 3.**
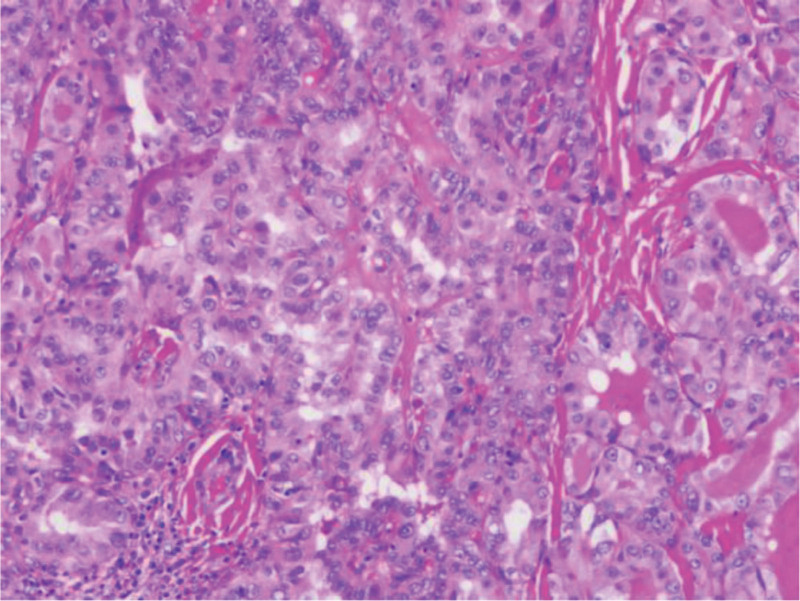
Postoperative Pathology showed apparent papillary structure throughout the tumor. The tumor was arranged in a papillary shape, with crowded and overlapping nuclei, ovoid, ground glass-like, with obvious nucleoli, and nuclear grooves and intranuclear pseudo-inclusions were seen in some cells (HE stain, original magnification, ×100).

Patients who completed the FNAB and CEUC examinations underwent total, subtotal, or partial thyroidectomy under general anesthesia, depending on the specifics of their thyroid masses. Postoperative specimens were stained with HE for pathologic diagnosis, and postoperative pathologic diagnosis was collected (Fig. 3).

### 2.5. Statistical analysis

All nodules were diagnosed by postoperative pathology as the gold standard. All data were statistically analyzed using SPSS23.0 software, and the counting data were tested by χ^2^ or Fisher’s exact test, and *P* < .05 was regarded as the difference was statistically significant. Statistical analysis: accuracy, sensitivity, and specificity of CEUS and FNAB diagnosis.

## 3. Results

Postoperative thyroid pathology was used as the final confirmation of the diagnosis into benign and malignant, and patients who had undergone both FNAB and CEUS were selected.

### 3.1. Comparison of the diagnostic efficacy of FNAB versus CEUS in thyroid nodules < 1 cm in maximum diameter

Comparison of results: The nature of the nodules was malignant in 84 cases and benign in 7 cases, as confirmed by surgical pathology results (Table 1). There were no complications such as hemorrhage and hematoma during and after the US-FNAB puncture. US-FNAB examination was positive in 65 cases and negative in 26 cases. CEUS examination was positive in 83 cases and negative in 8 cases.

**Table 1:** Comparison of thyroid nodules by US-FNAB, CEUS and surgical pathology in 91 patients.

Method	Inspection results	Postoperative pathology confirmed/case		Sensitivity (%)	Specificity (%)	Accuracy (%)	Positive predictive value (%)	Negative predictive value (%)
		Malignant	Benign					
CEUS	Negative	2	6	93.89	66.67	93.28	98.80	75.00
	Positive	82	1					
US-FNAB	Negative	19	7	87.02	100.00	87.31	100.00	26.92
	Positive	65	0					

CEUS = contrast-enhanced ultrasound, US-FNAB = ultrasound-guided fine needle aspiration biopsy.

In all cases, the sensitivity of CEUS diagnosis was 93.89% (82/84), the specificity was 66.67% (6/7), the accuracy was 93.28% (88/91), the positive predictive value was 98.80%, and the negative predictive value was 75.00%; the sensitivity of US-FNAB diagnosis was 87.02% (65/84), the specificity was 100.00% (7/7), accuracy was 87.31% (72/91), positive predictive value was 100.00% and negative predictive value was 26.92% (Table 1).

### 3.2. Comparison of diagnostic results of US-FNAB and CEUS for thyroid nodules with different diameters

Of the 110 thyroid nodules, all nodules were < 3 cm, of which 37 were < 0.5 cm in diameter, 54 were 0.5 to 1 cm in diameter, and 19 were ≥ 1 cm in diameter. Of the 112 thyroid nodules, there were 81 malignant nodules with cytologic diagnostic results from US-FNAB and postoperative pathological diagnostic results that were compatible with the diagnostic results of malignancy, of which 26 were < 0.5 cm in diameter, 39 were 0.5 to 1 cm in diameter, and 16 were ≥ 1 cm in diameter. Among 112 thyroid nodules, the number of malignant nodules with compatible CEUS diagnostic results and postoperative pathologic diagnostic results was 96, including 35 patients with a diameter of < 0.5 cm, 47 patients with a diameter of 0.5 to 1 cm, and 14 patients with a diameter of ≥ 1 cm. The sensitivity, specificity, and accuracy of the US-FNAB versus CEUS examination are detailed in Table 2.

**Table 2:** Comparison of diagnostic results of US-FNAB and CEUS for thyroid nodules with different diameters.

Method	Maximum diameter of the nodule (cm)	Inspection results	Postoperative pathology confirmed/case		Sensitivity (%)	Specificity (%)	Accuracy (%)
			Malignant	Benign			
CEUS	<0.5	Negative	0	2	100	100	100
		Positive	35	0			
	0.5–1	Negative	2	4	95.92	80.00	94.44
		Positive	47	1			
	≥1	Negative	4	1	77.78	100	78.95
		Positive	14	0			
US-FNAB	<0.5	Negative	9	2	74.29	100	75.68
		Positive	26	0			
	0.5–1	Negative	10	5	79.59	100	81.48
		Positive	39	0			
	≥1	Negative	2	1	88.89	100	89.47
		Positive	16	0			
Total			102	8			

CEUS = contrast-enhanced ultrasound, US-FNAB = ultrasound-guided fine needle aspiration biopsy.

The difference between the sensitivity of US-FNAB and CEUS examination was statistically significant (*P* < .05) in patients with diameters < 0.5 cm and 0.5 to 1 cm, and the difference between the sensitivity of US-FNAB and CEUS examination was not statistically significant (*P* > .05) in patients with diameters ≥ 1 cm (Table 3).

**Table 3:** Comparison of sensitivity between US-FNAB and CEUS (%).

Method	Maximum diameter of the nodule (cm)		
	<0.5	0.5–1	≥1
CEUS	100	95.92	77.78
US-FNAB	74.29	79.59	88.89
χ^2^	8.160	6.078	0.200
*P*	.004	.014	.655

CEUS = contrast-enhanced ultrasound, US-FNAB = ultrasound-guided fine needle aspiration biopsy.

## 4. Discussion

Thyroid nodules are one of the more common and frequent clinical diseases, and in recent years, with the development of imaging technology and people’s increased attention to their health, the detection and diagnosis rates of thyroid nodules have become higher and higher.^[2]^ Despite the favorable prognosis of PTC, lymph node metastasis and high recurrence rate may also occur in the early stage.^[14]^ Studies have shown that PTMC still has an lymph node metastasis rate of approximately 20%,^[15]^ and this rate is even higher for microcarcinomas with a maximum diameter close to 1 cm.^[5]^ Some patients developed local recurrence after surgery^[16]^ or even distant metastases to the lungs or brain.^[17,18]^ It has been shown that as the detection rate of thyroid nodules increases, patient anxiety leads to overtreatment, especially in patients with small thyroid nodules who are more likely to be overtreated, such as premature thyroidectomy, thyroid stimulating hormone (TSH) suppression therapy and iodine 131 therapy.^[2]^ Therefore, improving the diagnostic accuracy of small thyroid nodules will not only maximize the detection of early thyroid malignancy and improve patient prognosis with early surgical treatment but also reduce the overdiagnosis and overtreatment of benign nodules.^[19]^

US-FNAB is a safe, rapid, and accurate method that can be performed without anesthesia on an outpatient basis in a healthcare facility and is generally considered the “gold standard” for preoperative determination of the benign or malignant nature of thyroid nodules.^[6,7,20–22]^ However, the performance of US-FNAB in the diagnosis of thyroid nodules ≤ 1 cm in diameter is widely debated.^[23–25]^ The guidelines issued by the American Thyroid Association (ATA) in 2015 stated that for suspicious nodules < 1 cm detected by ultrasound without extra-thyroidal invasion or suspicious lymph node metastasis, close follow-up by the US is feasible and routine FNA,^[22]^ and some studies even advocate biopsy of nodules only 2.5 cm or larger.^[26]^ In the Japanese strategy for US diagnosis of thyroid nodules, FNAB is not recommended for nodules ≤ 5 mm, and should be considered in the presence of clinical lymphatic node metastasis, hoarseness due to laryngeal reentrant nerve (RLN) paralysis, or distant metastasis.^[27]^

Wu et al^[28]^ studied the results of US-FNAB in the diameter range of 4 to 10 mm thyroid nodules and showed that the sensitivity was 89.5%, specificity was 67.1%, and accuracy was 77.1% for small thyroid lesions < 1 cm in diameter. Sung et al^[29]^ found that FNA was sensitive, specific, and accurate for 555 thyroid nodules in group A (<10 mm) had a sensitivity of 67.8%, specificity of 100%, and accuracy of 77%; group B (≥10 mm) had a sensitivity of 69.4%, specificity of 100%, and accuracy of 85.7%, indicating that FNAB does not have a high diagnostic sensitivity and accuracy of <80% for PTMC nodules. In our study, the diagnostic sensitivity of US-FNAB was 87.02% (65/84), specificity was 100.00% (7/7), and accuracy was 87.31% (72/91) for the maximum diameter of thyroid nodules < 1 cm, suggesting that US-FNAB also has a better diagnostic efficacy for the diagnosis of thyroid micronodules.

The reasons for analyzing the large gap in the diagnostic results of US-FNAB in thyroid nodules ≤ 1 cm as described above are mainly because the results of US-FNAB are related to the experience and skill proficiency of the puncturing physician, and that improper or inadequate sampling can lead to unclear diagnosis, and that when the diameter of a thyroid nodule is ≤ 10 mm, the amount of cells in the specimen obtained is very small, making it difficult to analyze it.^[28]^ It is also related to factors such as the level of efficacy in diagnosing the indications of a suspicious cancerous nodule and the diagnostic level of the pathologist performing the cytology.^[23]^ Gross calcification within the nodule and necrotic liquefaction within the nodule affect the diagnostic efficacy of cytocentrifugal biopsy to some extent.^[28]^ In some studies, the diagnosis of US-FNAB was combined with the results of genetic testing, for example, the sensitivity of US-FNAB in diagnosing TMC was 96.03%, the specificity was 93.65%, and the accuracy was 95.24% in the study of Qin et al,^[23]^ whose study of US-FNAB combined with the results of the Braves genetic testing greatly improved the diagnostic accuracy of puncture. The results of US-FNAB combined with BRAF V600E gene detection reported in the study of Zhang et al^[30]^ were similar. In some studies, a secondary puncture was performed, which also improved the diagnostic accuracy of US-FNAB, for example, in a study by Kim et al,^[20]^ a secondary puncture was performed on unsatisfactory puncture specimens, and the sensitivity (87%), specificity (100%), and positive predictive value (100%) of US-FNAB were determined for the diagnosis of thyroid nodules with a maximal diameter of < 5 mm, negative predictive value (82%), accuracy (92%), false positive rate (0%) and false negative rate (8%). Although literature such as the above suggests that there are methods to improve the diagnostic efficacy of FNAB in small thyroid nodules, FNAB and genetic testing are expensive and few patients are willing to undergo a second puncture because FNAB is an invasive procedure.^[2]^ Reviewing the relevant literature, few articles investigated the application value of US-FNAB in the diagnosis of thyroid nodules with a maximum diameter of < 5 mm, and the results of our data: the accuracy rates of US-FNAB in the maximum diameter of < 0.5 cm, 0.5 to 1 cm, and ≥ 1 cm were 75.68%, 81.48%, and 89.47%, respectively, and the smaller the nodule, the diagnostic accuracy of US-FNAB the smaller the nodule, the lower the diagnostic accuracy of US-FNAB.

CEUS has been widely used in clinical practice as a noninvasive and economical screening modality. In a study by FENGSHENG LI et al, the positive detection rate of thyroid cancer by CEUS was higher than that of conventional US, with higher sensitivity and puncture point accuracy, and it also has great clinical application value in guiding thyroid puncture biopsy.^[31]^ Sun et al^[32]^ statistically analyzed 1003 patients with thyroid nodules who underwent CEUS, and the results showed that the diagnosis of thyroid nodules by ultrasonography thyroid nodules with a sensitivity, specificity, and ratio of 88%, 90%, and 63.18%, respectively. Yu et al^[33]^ did a meta-analysis including 597 thyroid nodules, and the sensitivity, specificity, and positive and negative likelihood ratios of CEUS for identifying benign and malignant lesions of the thyroid gland were 0.853, 0.876, 5.822, and 0.195, respectively. All of the above studies have shown that CEUS has high accuracy in diagnosing thyroid nodules, can be used as a good tool in the differential diagnosis of benign and malignant thyroid nodules, and has an important role in predicting the prognosis of patients with thyroid nodules.

There is now no consensus among researchers about the accuracy of CEUS in the diagnosis of thyroid nodules of different sizes. In a study by Li et al^[8]^ 185 small thyroid nodules with maximum diameters ≤ 1 cm and ≥ 0.5 cm in TR3-5 categories were subjected to CEUS characterization, and the sensitivity, specificity, accuracy, positive predictive rate, and negative predictive rate of CEUS 5-point characterization were 86.13%, 89.29%, 87.57%, 90.63%, and 84.27%, respectively. In Huang et al’s^[34]^ study, 109 patients with thyroid nodules with a maximum diameter ≤ 1 cm who underwent CEUS and surgery were collected and analyzed, and CEUS diagnosis of PTMC was found to be specific (62.50%) and sensitive (71.43%), suggesting that nodules with a maximum diameter > 0.66 cm can greatly improve the diagnostic accuracy of PTMC detection by CEUS.

In our study, the sensitivity of CEUS diagnosis of small thyroid nodules with a maximum diameter of ≥ 1 cm was 77.78%, the specificity was 100%, and the accuracy was 78.95%; the sensitivity of CEUS diagnosis of small thyroid nodules with a maximum diameter of 0.5 to 1 cm was 95.92%, the specificity was 80.00%, and the accuracy was 94.44%; and small thyroid nodules with a maximum diameter of < 0.5 cm The sensitivity of CEUS diagnosis was 100%, the specificity was 100%, and the accuracy was 100%. This indicates that CEUS has high accuracy in the diagnosis of small thyroid nodules. Unlike the above studies, our study showed that CEUS can still show good diagnostic accuracy in < 0.5 cm micronodules and has the best diagnostic efficacy in the micronodule group. In the 0.5 to 1 cm group, the postoperative pathology of the 1 case with false-positive CEUS showed Hashimoto’s thyroiditis, which was similar to papillary thyroid carcinoma in its heterogeneous low-enhancement state on CEUS and was difficult to distinguish on imaging, which is also consistent with previous studies.^[5]^

In a study comparing the diagnostic value of US-FNAB and CEUS at different sizes of thyroid nodule sizes, a study by Sun et al^[35]^ indicated that the sensitivity (81.25%) and specificity (85.71%) of US-FNAB for the diagnosis of TMCs was superior to the sensitivity (71.88%) and specificity (82.14%) of CEUS. In the diagnosis of TI-RADS category 4 thyroid nodules, a study by Liao et al^[36]^ showed that the sensitivity of US-FNAB and CEUS in patients with nodule diameter ≤ 10 mm was 87.50% and 100.00%, respectively, with a statistically significant difference (*P* < .05); among patients with nodule diameter > 10 mm, the sensitivity of US-FNAB and CEUS was 92.73% and 85.45%, the difference was not statistically significant (*P* > .05). Our findings were similar; for thyroid nodules < 10 mm in diameter, the sensitivity of CEUS examination (93.89%) was higher than that of US-FNAB (87.02%), and we recommend CEUS examination with high detection rate, safety, and economy; CEUS should be preferred for thyroid nodules < 0.5 cm in diameter (Table 3).

**Figure 4.**
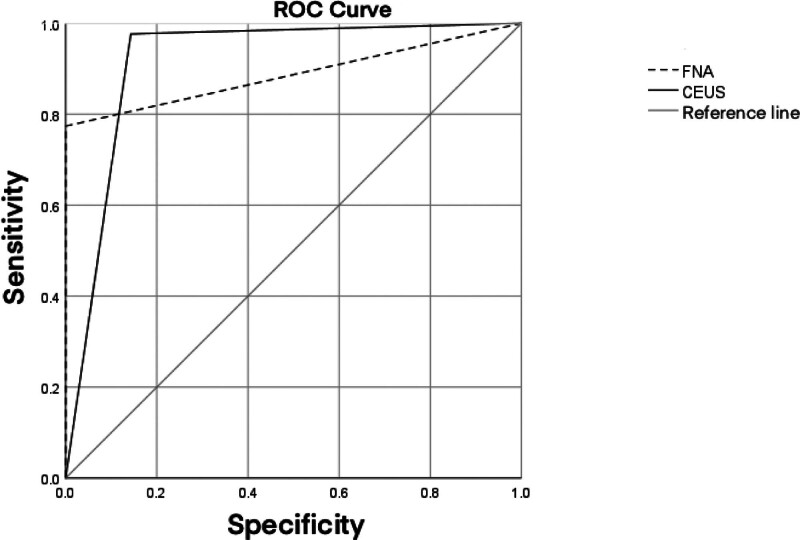
Comparison of receiver operating characteristic (ROC) analysis between FNAB and CEUS in the diagnosis of malignant thyroid nodules with a maximum diameter < 1 cm. CEUS = contrast-enhanced ultrasound, FNAB = fine-needle aspiration biopsy, ROC = receiver operating characteristic.

The sensitivity of CEUS examination was lower than that of US-FNAB examination in nodules ≥ 10 mm in diameter, probably due to the increased perfusion in malignant nodules ≥ 10 mm, and the malignant CEUS signs may overlap in both benign and malignant nodules; moreover, the diagnostic specificity of CEUS was lower than that of US-FNAB for thyroid nodules in our study, which may be attributed to the fact that nodular goiter in benign nodules with fibrosis or Hashimoto’s nodules presenting with fibrous tissue proliferation and lack of blood supply internally, resulting in a perfusion pattern similar to the contrast perfusion pattern of malignant nodules, thus showing inhomogeneous hypoenhancement and thus misdiagnosed as malignant nodules (Fig. 4).

In addition, the combined application of CEUS and FNAB has gained widespread attention in the clinic. It has been shown that CEUS improves the diagnostic efficiency of FNAB for high-risk thyroid nodules.^[37]^ Moreover, the combined application of the 2 modalities also has good diagnostic efficacy in other diseases, for example, translymphatic CEUS combined with sentinel lymph node FNAB has high accuracy in the preoperative assessment of axillary lymph node status,^[38]^ and in patients with pancreatic masses, Contrast-enhanced harmonic endoscopic ultrasound-guided FNAB is superior to standard Endoscopic ultrasound-guided FNAB.^[39]^ This integrated approach utilizes the dynamic imaging capabilities of CEUS to accurately guide fine-needle puncture and aspiration locations. Through the use of microbubble contrast, CEUS helps to depict the vascular architecture within the target, thus providing information on tissue blood flow that is critical for fine-needle puncture point selection and aspiration location, thus enhancing the accuracy of sample collection. The fusion of these 2 modalities significantly improved the diagnostic efficacy of the acquired cytology specimens and facilitated a more precise diagnosis, which could significantly improve diagnostic sensitivity, accuracy, and sample adequacy. The combined application of the 2 modalities requires further investigation.

## 5. Conclusion

For thyroid nodules ≤ 1 cm in diameter, the sensitivity and specificity of CEUS examination are higher than that of US-FNAB; and CEUS still has better diagnostic accuracy in the diagnosis of benign and malignant thyroid nodules < 0.5 cm, CEUS is recommended for thyroid nodules diagnosed negatively by US-FNAB and < 1 cm in diameter; and CEUS should be preferred for thyroid nodules < 0.5 cm in diameter.

## Author contributions

**Data curation:** Yuxia Yang.

**Funding acquisition:** Liquan Ouyang.

**Investigation:** Qi Liu.

**Methodology:** Qi Liu.

**Software:** Liquan Ouyang.

**Writing – original draft:** Qi Liu.

**Writing – review & editing:** Shengchu Zhang.
